# Oral immunotherapy with peach juice in patients allergic to LTPs

**DOI:** 10.1186/s13223-019-0374-x

**Published:** 2019-09-24

**Authors:** Begoña Navarro, Eladia Alarcón, Ángela Claver, Mariona Pascal, Araceli Díaz-Perales, Anna Cisteró-Bahima

**Affiliations:** 1 Allergy Department, Hospital Universitari Dexeus (GQS), Universitat Autònoma de Barcelona, Calle Sabino Arana, 5-19, 08028 Barcelona, Spain; 2 Servicio de Inmunología, Centro de Diagnóstico Biomédico, Hospital Clinic, Universitat de Barcelona, Barcelona, Spain; 3 Escuela Técnica Superior de Ingeniero Agrónomos, Departamento de Biotecnología, Universidad Politécnica de Madrid, Madrid, Spain

**Keywords:** Anaphylaxis, Sublingual immunotherapy, Peach, Allergy

## Abstract

**Introduction:**

To assess the safety and efficacy of an oral immunotherapy regimen in patients with allergy to lipid transfer proteins (LTPs).

**Materials and methods:**

Prospective study of 24 patients allergic to LTP with positive skin test and a history of anaphylaxis. All patients underwent a desensitization protocol with commercial peach juice. Rising doses of peach juice were administered, starting with an initial dose of seven drops of a 1/1000 dilution and finishing with a dose of 5 ml at visit 17. At visit 18, all patients performed an open challenge with whole juice at a cumulative dose of 200 ml. All adverse reactions occurring during the administration of the different doses were recorded. Levels of rPru p 3 in the juice were quantified.

**Results:**

There were no severe reactions during the desensitization process in the 24 patients. Seven patients (29%) reported mild oral symptoms, and two patients (8%) had urticaria associated with co-factors (one due to exercise and another due to non-steroidal anti-inflammatory drugs). Nineteen patients were able to swallow 5 ml of juice and five withdrew from the study. In two pregnant patients the final challenge was not performed. In all, 17/24 patients were able to consume 200 ml peach juice without developing symptoms.

**Conclusions:**

Oral immunotherapy with the regimen used in this study is an effective and safe short-term therapeutic option for patients with allergy to LTPs. Commercial peach juice appears to be suitable for this treatment.

## Introduction

Lipid transfer proteins (LTPs) are the primary cause of food allergy in the adult population in the Mediterranean area [1, 2]. LTPs are a family of proteins that are widely distributed in the plant kingdom [3], and are found in the pollen of trees, plants and latex. The fruit most widely implicated in this allergy is the peach [4, 5].

Oral immunotherapy (OIT) to food consists of the oral administration of the food allergen causing the symptoms, starting with minimal amounts and progressively increasing until reaching the normal amount ingested according to the subject’s age or the maximum tolerated threshold dose [6]. The possibility of inducing desensitization to peach LTPs has given rise to several studies [7–12] in which commercial products with a known quantity of this protein are developed to allow the design of sublingual desensitization protocols [10, 12].

After several months of sublingual immunotherapy (SLIT), many patients who previously presented positive skin tests or anaphylaxis to certain foods due to their LTP content are able to consume these foods without adverse reactions [10]. However, since 2015, difficulties in obtaining the fresh fruit required to extract the LTPs have limited the possibility of carrying out SLIT.

Based on the OIT protocols for cow and egg milk proteins developed by our group [13, 14], we describe an OIT protocol using fresh product in patients allergic to LTPs. Due to the difficulty of obtaining fresh peaches throughout the year, commercial peach juice packaged in cartons was used. The brand (Granini) uses fruit obtained from an area of Italy with a high percentage of residents allergic to LTP.

## Materials and methods

### Study population

This prospective study carried out from January 2015 to October 2016 included 24 patients allergic to LTP with a positive skin test to a 0.1 mg/ml concentration of LTP (Bial-Aristegui^®^, Bilbao, Spain) and a history of anaphylaxis to peach (Table 1). Twenty-one (87%) patients also presented anaphylaxis after ingesting nuts and, in three cases, anaphylaxis reactions also occurred after the intake of lettuce, plum and/or cherry. In addition, all patients manifested minor symptoms (oral allergy syndrome, mild urticaria) after ingestion of other foods such as lettuce and rosacea fruits. The anaphylactic reaction in all patients was Grade II or III, with the involvement of more than one organ [15]. We excluded patients who were sensitized to LTP without symptoms, pregnant patients, patients with severe immune and/or cardiovascular diseases and/or patients receiving treatment with beta blockers. Patients with poor treatment compliance and patients who do not understand the aim of the study were also excluded. The study was approved by the Center’s Ethical Committee (act of approval 54/2018) and the patients provided signed informed consent prior to recruitment.

**Table 1:** Demographic characteristics of the study population

Sex, male, n	7
Age (mean and range), years	25.5 (5–42)
Clinical symptoms (anaphylaxis)	24 (9 associated with a cofactor: 4 exercise and 5 NSAIDs)

Specific IgE	n (%)	kU/L^a^
Pru p 3	24 (100)	7.43 (0.40–100)
Peach	24 (100)	6.77 (1.00–76.10)
Green bean	6 (25)	3.44 (0.44–71.00)
Almond	8 (33)	2.91 (0.41–7.57)
Hazelnut	10 (42)	2.53 (0.62–16.30)
Nut	8 (33)	16.00 (2.44–42.90)
Peanut	16 (67)	1.50 (0.42–22.70)
Lettuce	6 (25)	2.54 (0.58–20.00)
Apple	7 (29)	2.64 (0.59–32.00)
Chickpea	2 (8)	11.79 (3.28–20.30)
Plum	3 (12)	3.23 (1.60–30.10)
Lentil	3 (12)	1.26 (1.06–21.50)
Sunflower pip	3 (12)	10.70 (8.00–10.88)
Grape	3 (12)	2.28 (0.59–3.54)
Wheal diameter prick-test LTP (mm)^a^	7.75 (3.00–11.50)	
Wheal diameter prick-by-prick Granini^®^ juice (mm)^a^	6.00 (3.50–11.00)	
Prick-by-Prick Granini^®^ juice dilutions at end point	1/100 (6 patients)	
	1/10 (14 patients)	
	1/1 (4 patients)	
Prior ALK-Abelló^®^ peach SLIT, n (%)	7 (29%)	

*NSAIDs* nonsteroidal anti-inflammatory drugs, *LTPs* lipid transfer proteins, *SLIT* sublingual immunotherapy

^a^Data are expressed as median (range)

All patients underwent a detailed medical history, including information on atopy and previous symptoms of respiratory allergy, as well as the clinical symptoms, causative foods, and possible cofactors involved in the reaction.

### Skin tests

Skin tests were performed in the forearm, with a battery of pneumoallergens including profilin (ALK-Abelló^®^, Madrid, Spain) and LTP (Bial-Aristegui^®^, Bilbao, Spain), in addition to the foods involved in the reaction. Tests which presented wheals equal to or larger than histamine-induced wheals were considered positive. Prick-by-prick tests were performed with commercial peach juice at different dilutions to establish the “end point”. The dilutions of the juice used to carry out the tests were 1/10, 1/100 and 1/1000. Skin prick tests with these samples were performed in order to determine the safest dilution for the patient to start the OIT regimen (end point). The starting dose in the challenge was the one with a wheal area smaller than the histamine-induced wheal [16].

### Measurement of specific IgE

Total IgE, baseline tryptase and specific IgE to the foods involved were measured. Levels of specific IgE to Pru p 3 were also quantified. The responses to the prick test with LTP (Bial-Aristegui^®^) and the prick-by-prick test with juice were recorded at each dose increase.

### Source of the allergen

The source of the allergen used for the desensitization protocol was a commercial peach juice (Granini, Eckes Granini Iberica SA, Barcelona) which uses the Percoca peach variety, from Italy. The stability of rPru p 3 in the juice was analysed by prick-by-prick test up to 1–2 weeks after the carton was opened.

Agarose gel electrophoresis analysis of the commercial peach juice was performed and the levels of rPru p 3 were quantified. For this quantification, 1 ml of the juice was precipitated with 10% TCA for 1 h at 4 °C. The precipitate obtained was washed extensively with cold acetone. After drying the precipitate, it was resuspended in Phosphate buffered saline (PBS) and used to coat Enzyme-Linked ImmunoSorbent Assay (ELISA) plates. After blocking, the samples were incubated with antibody produced against Pru p 3 (1:1000) and developed with peroxidase-bound secondary antibody. The amount of LTP contained in the sample was determined by extrapolation of values to that obtained by a standard curve with known values of Pru p 3. The determination was made using three different batches of juice, and repeated three times in each batch.

### Sublingual specific immunotherapy (desensitization protocol)

Table 2 shows the desensitization protocol used in the study. The OIT was performed in four stages divided into 18 visits at our unit. Increasing doses of peach juice were administered, starting with an initial dose of seven drops of a 1/1000 dilution until reaching a dose of 5 ml at visit 17. During the first seven visits, the dose was administered sublingually and was kept in the mouth for 2 min without being ingested. Subsequently, patients maintained this dose at our unit and also daily at home, before returning to our unit to increase the dose after 7–15 days. From visit 8 onwards patients began to swallow the drops, starting with one undiluted drop (1/1) until reaching a dose of 5 ml by visit 17. Before each dose increase, the skin tests were repeated with LTP (Bial-Aristegui^®^, Bilbao, Spain), with the commercial peach juice which patients returned after administration at home and with the new carton of juice open for the dose increases. Finally, at visit 18, all patients underwent no symptoms with the whole juice until reaching a cumulative dose of 200 ml. This amount was subsequently maintained at home, either daily or at least three times a week. All adverse reactions occurring during the administration of the various doses were recorded.

**Table 2:** OTI guidelines with Granini^®^ peach juice

Stage 1. Sublingual 2 min and then spit out	
Visit 1 (dilution 1/1000)	1–2–4 drops
Visit 2 (dilution 1/100)	1–2–4 drops
Visit 3 (dilution 1/10)	1–2–4 drops
Visit 4 (1/1: undiluted juice)	1 drop
Visit 5 (1/1)	2 drops
Visit 6 (1/1)	4 drops
Visit 7 (1/1)	5 drops
Stage 2. Sublingual 2 min and swallow (drops)	
Visit 8 (1/1)	1 drop
Visit 9 (1/1)	2 drops
Visit 10 (1/1)	4 drops
Visit 11 (1/1)	5 drops (equivalent to 0.13 ml)
Stage 3. Sublingual 2 min and swallow (ml)	
Visit 12	0.25 ml
Visit 13	0.50 ml
Visit 14	1 ml
Visit 15	2 ml
Visit 16	3 ml
Visit 17	5 ml
Stage 4. Oral tolerance test with whole juice	
Visit 18	10–25–65–100 ml (accumulated dose 200 ml)
Continue intake of 200 ml/day (1 carton), at least 4 days/week	

The increases were performed in a controlled manner at the immunotherapy unit every 1, 2 or every 4 weeks, depending on tolerance. Previously, prick-test to LTP and prick-by-prick test to juice were repeated (with the used and the new cartons), to assess the possible loss of protein activity of the product administered at home

The final goal was for patients to ingest a serving-sized amount of peach, either 200 ml of peach juice (the size of the juice box) or a serving of fresh peach. If for any reason this figure was not reached, oral food challenge was considered satisfactory if the patient did not present adverse effects with the dose reached and if the challenge with peach was well tolerated.

## Results

Twenty-four patients were included, aged between 5 and 42 years (seven males) and diagnosed with anaphylaxis due to allergy to peach (Table 1) in the 3 months preceding their inclusion in the study. In nine patients, co-factors were present in the anaphylactic reactions (exercise in four and use of non-steroidal anti-inflammatory drugs, NSAIDs, in five). At the time of diagnosis, all the patients presented positive skin tests against LTP (Bial-Aristegui^®^) and specific IgE to rPru p 3. Seven patients (29%) had previously undergone peach SLIT with a commercial extract (ALK-Abelló^®^, Madrid, Spain) for 4 to 18 months. At the time of the study, these patients had been untreated for a period of 4–6 months. No differences in the initial dilution were found between patients with a history of treatment with ALK peach SLIT and those without.

**Fig. 1:**
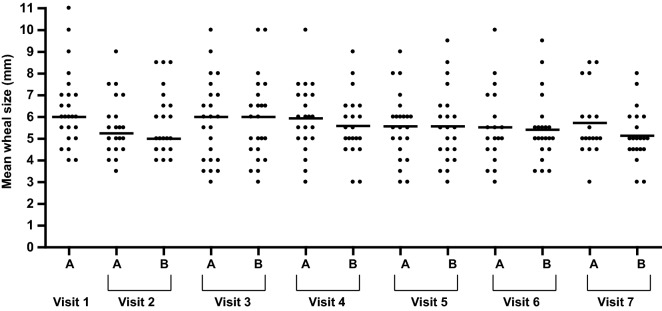
Prick-by-prick test performed either with the juice just opened (**a**) or with the juice returned by the patient within 8-15 days of taking the doses at home (**b**)

No significant differences were found in the wheals produced by the prick-by-prick test performed either with the juice just opened or with the juice returned by the patient within 8–15 days of taking the doses at home (Fig. 1).

**Fig. 2:**
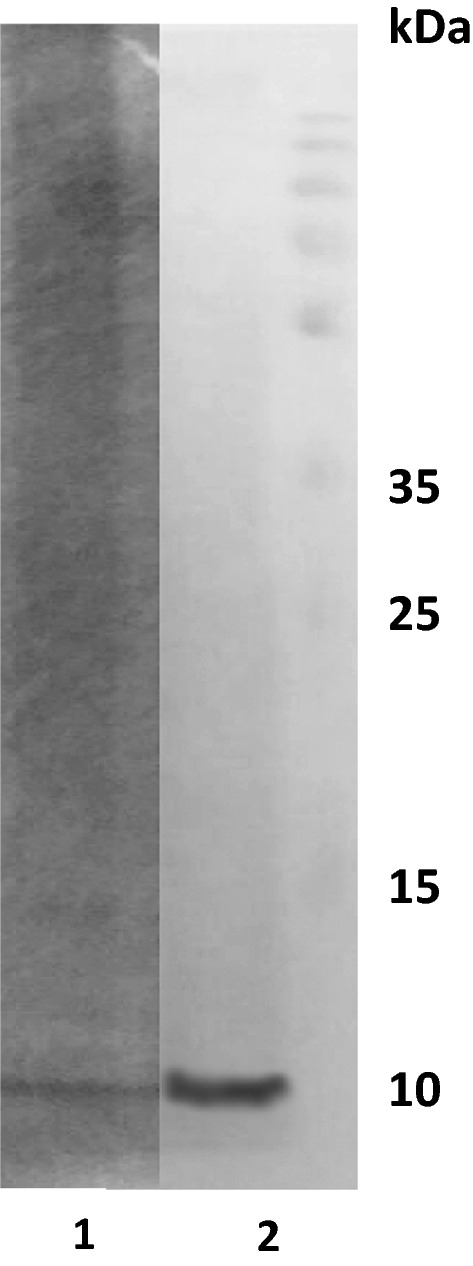
Agarose gel electrophoresis with Granini^®^ commercial peach juice: 10 μg/2 ml precipitate with TCA 20%. 1. Coomassie staining; 2. Anti-Pru p 3 (1:1000). A band of 10 kDa is visible in lane 2

The agarose gel electrophoresis analysis of the peach juice showed a band of 10 kDa that was identified as Pru p 3 (Fig. 2). Quantification by ELISA showed that the commercial juice had an LTP concentration of 21.16 μg/ml (Table 3).

**Table 3:** Dose of Pru p 3 in peach juice vs sublingual peach immunotherapy

	Pru p 3 µg/ml	Pru p 3 dose: 5 drops at maximum concentration (µg) (5 drops = 0.13 ml)
ALK juice SLIT	50	6.5 (jar 4: maximum concentration)
Granini^®^ peach juice	21.16	2.75

*SLIT* sublingual immunotherapy

The desensitization pattern was started with the dilution of peach juice prior to the one that induced a wheal equal to the histamine-induced wheal (end point). The desensitization regimen was initiated with the 1/100 dilution in six patients (one of whom had previously been treated with ALK-Abelló^®^ peach SLIT); 14 patients started desensitization with a 1/10 dilution (five previously treated with ALK-Abelló^®^ peach SLIT); four patients started the protocol with a 1/1 dilution (one previously treated wth ALK-Abelló^®^ peach SLIT). From that point onwards, the doses continued to increase in accordance with the protocol (Table 2).

There were no serious reactions during the desensitization process; none presented any serious reactions during the process. Fifteen patients out of 24 (63%) presented no reaction of any kind, 7/24 (29%) reported mild oral symptoms, which remitted immediately with the intake of cold water, and finally 2/24 (8%) had urticaria after taking the dose at home, associated with co-factors (exertion, non-steroidal anti-inflammatory drugs).

**Fig. 3:**
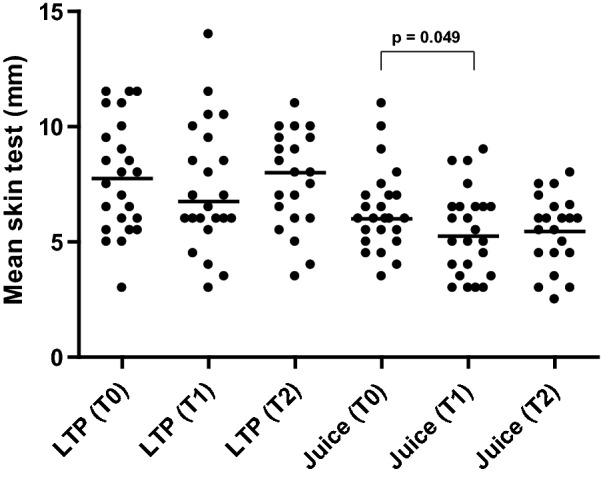
Skin test with LPT and with Granini^®^ commercial peach juice: T0—before stage 1; T1—after stage 1; T2—after stage 3

As regards cutaneous reactivity, the size of the wheals in the skin tests performed with commercial peach juice decreased between visits T0 and T1 (p = 0.049), but there was no reduction in the size of the wheals in the skin tests with LTP (Bial-Aristegui^®^) in none of the intervals or between visits T0 and T2 in the commercial peach juice (Fig. 3).

Desensitization was induced in 19/24 (79%) of patients. Fourteen patients were able to swallow 200 ml of commercial juice and in three patients the direct challenge test with peach presented no symptoms; in two pregnant patients the final challenge test was not performed but they were able to maintain the dose of 5 ml swallowed at home. Five patients withdrew from the study. The mean time taken to swallow 200 ml (T4: visit 18 of the protocol) was 3.6 months.

In most cases, the treatment allowed patients to ingest foods which had previously been absent from their diet, without presenting symptoms. Patients were encouraged to maintain regular intake of the maximum doses reached and to avoid co-factors.

## Discussion

Food allergy negatively affects patients’ quality of life, obliging them to avoid certain foods that are widely consumed in their environment. In order to remedy this situation, treatments such as OIT have been introduced over the last decade or so and have achieved success rates ranging between 40 and 90% [17–19].

The peach is the fruit most frequently involved in food allergic reactions in the adult population in the Mediterranean area [2, 5]. The severity of this allergy is probably related to the high stability of its major allergen, Pru p 3 (which is stable to heat and digestion with pepsin) [20]. Sensitization to the LTP Pru p 3 has been observed in more than 90% of patients with peach allergy in the Mediterranean area [21].

The present study demonstrates that OIT is possible in patients with allergy to LTPs, using a commercial peach juice containing LTP as the source of the allergen. Previous work has demonstrated that desensitization is possible; however, extracts are not uniformly accessible. The current study offers a safe and effective desensitization protocol using a widely available and cost-effective commercial juice, which can improve patients’ access to this important therapy [8–10]. As the LTP concentration of the juice is unknown a priori, the trial simulates the situation in real life since we do not know the LTP concentration of the fruit and vegetables consumed by our patients. After OIT, most of our patients were able to ingest up to 200 ml of juice after 18 visits; there are few adverse reactions, and the skin reactivity to the juice is reduced. In addition, the protocol has proved to be both safe and effective: no severe reactions were recorded during the protocol and all patients presented no symptoms when swallowing the juice.

The main limitation of this study is that it is an initial pilot study. However, its strength is that the desensitization protocol is carried out with the same product and using the end point to establish the initial and follow-up dose, as well as the final challenge. In addition, the study allowed us to define the behavior of a group of patients during OIT with peach juice, and the results obtained will enable us to carry out further double-blind studies. Another limitation of the study is that we cannot talk about the development of tolerance, because there was no temporary avoidance period preceding the challenge procedure. Future studies should aim to explore the possible development of tolerance with this desensitization protocol. Finally, the lack of a baseline challenge, particularly in those patients whose reactions occurred following dual exposure to peach and a cofactor, may be an additional limitation.

In conclusion, the study demonstrates that the current protocol using a cost-effective and accessible commercial peach juice is safe and effective for desensitizing allergic patients sensitized to the peach LTP Pru p 3.

## Data Availability

Yes

## Abbreviations

ELISA: Enzyme-Linked ImmunoSorbent Assay
LTPs: lipid transfer proteins
NSAIDs: non-steroidal anti-inflammatory drugs
OIT: oral immunotherapy
PBS: phosphate buffered saline
SLIT: sublingual immunotherapy
